# The Rights of the Civil Population

**Published:** 1914-08-29

**Authors:** 

**Affiliations:** Kenwood, Hampstead, N.W., President


*To the Editor of* THE HOSPITAL.

### The Rights of the Civil Population.

*To the Editor of* THE HOSPITAL.

SIR,—As President of the Hampstead General Hospital, I desire to inform you that the Council have placed at the disposal of the Government forty beds for the use of the sick and wounded in the war.

To enable the hospital to meet the demands made upon its resources by the sick poor of the district, which are exceptionally heavy, it is essential that the funds, now at a very low ebb, should be considerably augmented. In these circumstances I venture to appeal to the public to support the Council in their efforts to maintain the work of this hospital.—I am, yours faithfully,

GRAND DUKE MICHAEL,

Kenwood, Hampstead, N.W., President.

[This letter has more significance than its face value, for it points to the fact that the prime duty of the voluntary hospitals and their staffs is to the civil population. It also points to the fact that as one of the concomitants of the war general sickness is likely to increase, and that therefore to deplete the hospitals of their staffs unduly—or their funds—is the worst and most thoughtless sort of patriotism.—ED. THE HOSPITAL.]

